# Identification and validation of uterine stimulant methylergometrine as a potential inhibitor of caspase-1 activation

**DOI:** 10.1007/s10495-017-1405-z

**Published:** 2017-07-28

**Authors:** Guillermo García-Laínez, Mónica Sancho, Vanessa García-Bayarri, Mar Orzáez

**Affiliations:** Laboratory of Peptide and Protein Chemistry, Centro de Investigación Príncipe Felipe, C/ Eduardo Primo Yúfera, 3, 46012 Valencia, Spain

**Keywords:** Caspase-1 inhibitor, Ergometrine, Inflammasome inhibitor, Inflammation, Methylergometrine

## Abstract

Inflammasomes are intracellular multiprotein complexes of the innate immune system. Upon an inflammatory insult, such as infection or intracellular damage, a nucleotide-binding oligomerization domain-like receptor (NLR) sensor protein and the adaptor protein ASC (apoptosis-associated speck-like protein containing a caspase activation and recruitment domain) are assembled to activate protease procaspase-1. This protease processes pro-IL-1β and pro-IL-18 cytokines, which are released to induce the inflammatory response. De-regulation of inflammasome contributes to the progression of several diseases, such as Alzheimer’s disease, diabetes, cancer, inflammatory and autoimmune disorders. We herein describe the identification of methylergometrine (MEM), a drug currently used as a smooth muscle constrictor during postpartum hemorrhage, as an inhibitor of the inflammasome complex in ASC-mediated procaspase-1 activation screening. MEM inhibits the activation of the nucleotide-binding oligomerization domain-like receptor protein 1 (NLRP1) and nucleotide-binding oligomerization domain-like receptor protein 3 (NLRP3) inflammasomes in cellular models upon different pro-inflammatory stimuli. Our results suggest that MEM has the potential to reposition in the treatment of inflammatory diseases with the advantages of established safety and clinical data.

**Electronic supplementary material:**

The online version of this article (doi:10.1007/s10495-017-1405-z) contains supplementary material, which is available to authorized users.

## Introduction

Inflammation is a protective response of the body to ensure removal of detrimental stimuli and repair damaged tissue [1]. Cells of the innate immune system, such as macrophages and dendritic cells, recognize the conserved microbial structures called pathogen-associated molecular patterns (PAMPs) and danger-associated molecular patterns (DAMPs) [2]. At least four major pattern recognition receptor (PRR) families operate cooperatively to detect invading microbes or endogenous danger signals: Toll-like receptors, RIG-I-like receptors, C-type lectin receptors, and nucleotide-binding oligomerization domain leucine-rich repeat containing receptors (NLRs) [3, 4]. After microbial recognition, PRRs induce the activation of different signaling pathways that result in the synthesis of pro-inflammatory cytokines such as interleukin (IL)-1β (IL-1β) and IL-18 [5]. IL-1β and IL-18 are synthesized as precursor molecules, which can be cleaved into their mature and active forms by caspase-1 protease. This activation occurs when the cell receives a second signal (PAMP or DAMP) to activate NLRs and promotes the assembly of a large multiprotein complex called the inflammasome. Inflammasomes are a group of multimeric protein complexes formed by the NLR sensor protein, the adaptor protein ASC (the apoptosis-associated speck-like protein containing a caspase activation and recruitment domain (CARD)) and protease procaspase-1 (PC1). ASC interacts through the pyrin domain with sensors, and through the CARD with PC1. The formation of this complex leads to the proximity-induced activation of PC1 [6]. Once activated, caspase-1 hydrolyses the inactive forms of pro-inflammatory cytokines IL-1β and IL-18 (pro-IL-1β and pro-IL-18) by triggering their secretion and initiating the inflammation process. The most reported consequence of caspase-1 activation is the rapid secretion of cytokines, but caspase-1 activation in macrophages also leads to caspase-1-dependent cell death called pyroptosis [7–9].

Abnormal inflammasome activation has been implicated in the pathophysiology of Alzheimer’s disease [10, 11], diabetes [12] and cancer [13, 14], and also leads to many inflammatory and autoimmune diseases, including gout, silicosis, rheumatoid arthritis and genetically-inherited periodic fever syndromes [15–18].

The identification of small molecule inhibitors that target the inflammasome is an important step in developing effective therapeutics to treat inflammation. Recently developed treatments for inflammasome-related diseases target processes downstream of PC1 activation. These include Anakinra, a recombinant IL-1 receptor antagonist [19, 20], Canakinumab, a neutralizing IL-1β antibody [21, 22], and Rilonacept, a soluble decoy IL-1 receptor [23]. Several relevant strategies have also identified new drugs that target different intervention points in the cascade of inflammasome activation, such as MCC950 [24], Glyburide [25], β-hydroxybutyrate [26] and VX-765 [27].

In this study, we address the identification of new modulators of the inflammasome by targeting ASC-mediated PC1 activation and employing a drug repurposing strategy. The major advantage of drug repurposing is that it saves the time and cost for drug development, and permits the rapid inclusion of the molecule in clinical trials [28, 29]. We describe the identification of methylergometrine (MEM), a synthetic analog of ergonovine prescribed for treating postpartum hemorrhage, as an inhibitor of ASC-mediated PC1 activation and demonstrate the inflammasome inhibition activity of this drug in cellular models of inflammation.

## Results

### Identification of modulators of the ASC-mediated activation of PC1

**Fig. 1:**
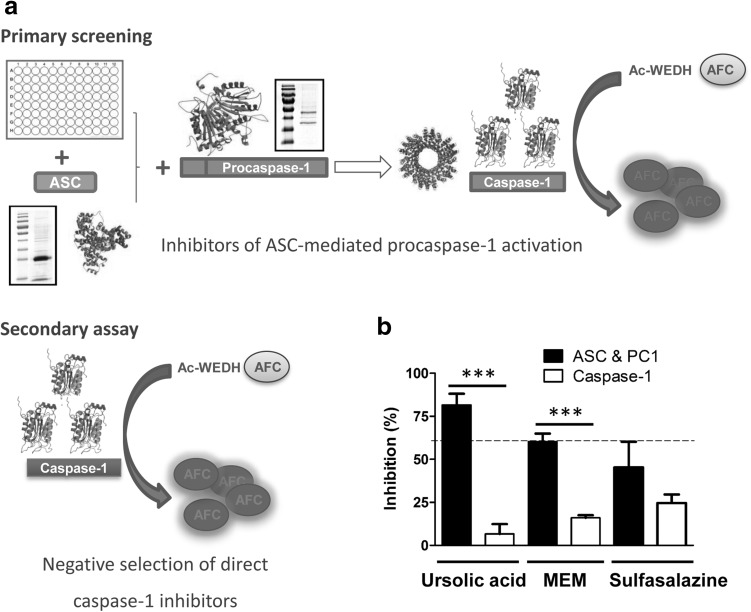
Identification of the modulators of the ASC-mediated activation of PC1. **a** Scheme of the 96-well plate assays performed to identify inhibitors. Primary screening: ASC-dependent PC1 activation was monitored in vitro by the release of AFC from fluorogenic PC1 substrate Ac-WEHD-AFC in the presence of 880 FDA-approved compounds (20 µM) from the Prestwick Chemical Library. Secondary screening: caspase-1 activation was monitored in vitro by the release of AFC from fluorogenic PC1 substrate Ac-WEHD-AFC in the presence of the positive hits (20 µM) obtained in the previous assay. **b** Graphical representation of the behavior of the three selected compounds in the primary (*black bars*) and secondary screening (*white bars*) at 20 µM in DMSO. A Student’s *t*-test statistical analysis (***p < 0.01) was performed to compare the caspase-1 inhibition for each compound in the presence or absence of ASC. Data represent the mean ± SD of three experiments

Identification of inflammasome inhibitors was accomplished by targeting the specific interaction of PC1 with the adaptor molecule ASC. The Prestwick Chemical Library^®^ composed of 880 FDA-approved drugs, selected for their chemical and pharmacological diversity and safety in humans, was evaluated in a caspase-1 activity fluorescence assay. Recombinant PC1 and ASC were combined to obtain a functional complex. Once reconstituted, self-cleavage produces PC1 activation. Active caspase-1 hydrolyzes substrate Ac-WEDH-AFC by releasing the fluorogenic AFC molecule (Fig. 1a). Compounds were pre-incubated with recombinant ASC to facilitate the selection of the molecules that affect the ASC/PC1 interaction interface. A 60% inhibition threshold was applied, and those compounds that showed intrinsic fluorescence or color were ruled out. Three compounds were initially selected as inhibitors of ASC-mediated PC1 activation: ursolic acid, MEM and sulfasalazine. A secondary screening was performed to confirm positive hits and to rule out direct inhibitors of active caspase-1 (Fig. 1a). Ursolic acid and MEM were selected as inhibitors of the ASC-mediated activation of caspase-1 (Fig. 1b).

**Fig. 2:**
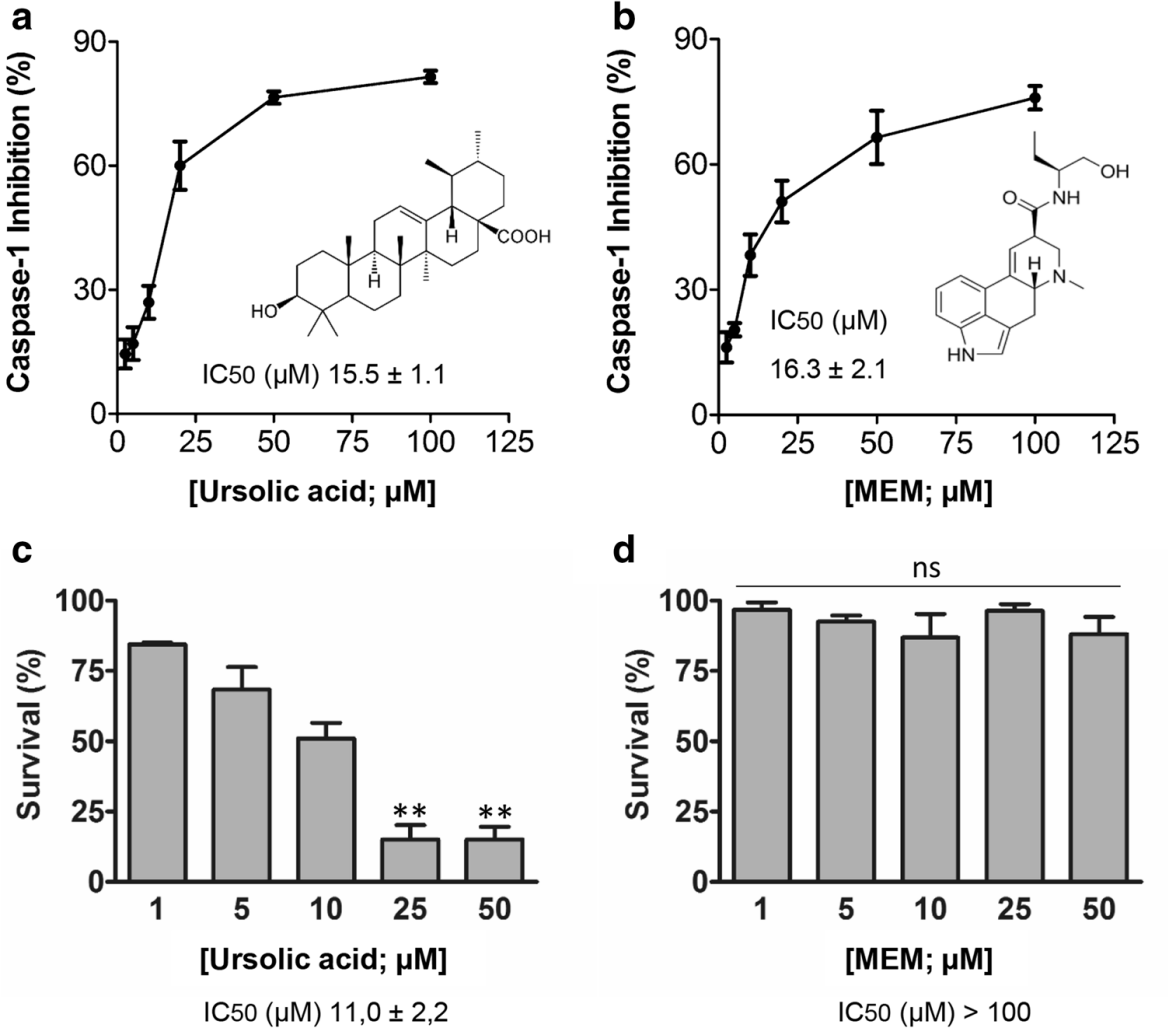
The activity and toxicity profiles of ursolic acid and MEM. Dose–response activity of ursolic acid (**a**) and MEM (**b**) in vitro. The inhibitory effect of both compounds on ASC-dependent PC1 activation was followed by incubating PC1 and ASC with different concentrations of each compound: ursolic acid (2.5, 5, 10, 20, 50 and 100 µM) and MEM (2.5, 5, 10, 20, 50 and 100 µM). Data are reported as the percentage of inhibition compared with the untreated sample. The concentration that provided 50% inhibition of the complex (IC_50_) was calculated. Data represent the mean ± SD of three experiments. Toxicity was evaluated in THP-1 cells. The cell viability measured by the MTS assay was monitored after 24 h of treatment with the indicated concentrations of both compounds, ursolic acid (**c**) and MEM (**d**). Data are reported as the percentage of live cells compared with the untreated sample. Data represent the mean ± SD of three experiments. Asterisks represent significant differences, as determined by a one-way ANOVA test with Dunnetts’s multiple *post test* comparison **p < 0.05, ns means non significant

In order to confirm ursolic acid and MEM as positive hits, the dose–response effect on the ASC-dependent PC1 activation assay was analyzed. In the presence of ursolic acid and MEM, activity was inhibited in a dose–response manner with an IC_50_ value (compound concentration that provides 50% inhibition of activity) within the low micromolar range (15.5 and 16.3 µM, respectively) (Fig. 2a, b). Both compounds were then evaluated in a cellular environment for toxicity. For this purpose, we employed THP-1 cells, a human monocyte-derived cell line frequently used as a mammalian cellular model for inflammasome activation studies. Ursolic acid was ruled out because of the high toxicity observed in THP-1 cells (Fig. 2c). However, MEM showed a non toxic profile at the range of concentrations from 1 to 50 µM (Fig. 2d).

### Anti-inflammatory capacity of MEM as an inhibitor of the ASC-mediated activation of PC1

**Fig. 3:**
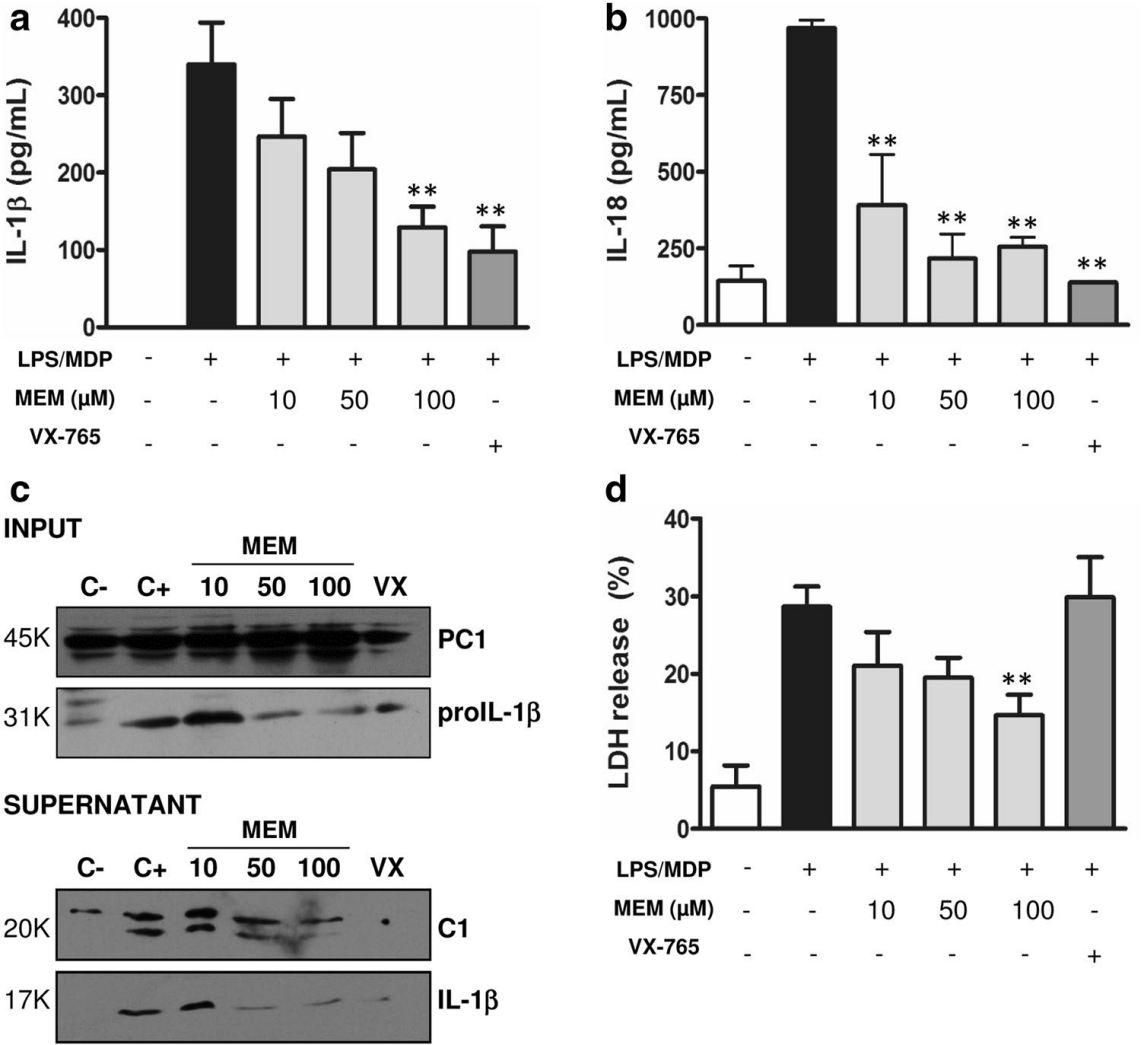
MEM inhibits the NLRP1-dependent inflammasome in THP-1 cells. IL-1β (**a**) and IL-18 (**b**) secretion was evaluated by the ELISA technique upon activation of the NLRP3 inflammasome with LPS (100 ng/ml) and MDP (50 µg/ml). Cells were treated with MEM at 10, 50 or 100 µM, and with VX-765 (VX) at 5 µM. **c** THP-1 cells were stimulated as described above, and supernatants and inputs were analyzed by Western blotting; IL-1β and cleaved caspase-1 (C1) were detected. The shown data are representative of three independent experiments. **d** Measurement of the release of LDH into the extracellular medium under the above-described conditions. C−: cells non-treated and C+: cells treated with stimuli activation but without the anti-inflammatory treatment. *Asterisks* represent significant differences to the positive control (C+), as determined by a one-way ANOVA test with Dunnetts’s multiple *post test* comparison **p < 0.05. All the data are expressed as the mean ± SD of three experiments

To investigate the anti-inflammatory properties of MEM, its activity was assayed in THP-1 cells upon different pro-inflammatory signaling. NLRP1 and NLRP3 are ASC-dependent inflammasomes [30], and thus appropriate cellular targets to evaluate identified inhibitors. The NLRP1 inflammasome was activated by using a combination of lipopolysaccharide (LPS) and muramyldipeptide (MDP) stimuli [31]. Then NLRP1 inhibition was evaluated at three different MEM concentrations. IL-1β and IL-18 secretion, cytotoxicity and PC1 activation were analyzed in all cases (Fig. 3). VX-765, a pro-drug that is undergoing clinical research which inhibits caspase-1 and reduces the release of IL-1β and IL-18, was used as a positive control of caspase-1 inhibition [27]. A significant inhibition of IL-1β and IL-18 release were detected at 100 µM of MEM (Fig. 3a, b). In order to confirm these results obtained in the ELISA assays, immunoblotting of cellular supernatants was also performed. A significant inhibition of caspase-1 and IL-1β cleavage was observed at the highest MEM dose (Fig. 3c and Supp Data 1). The observed drop in proIL-1β in the MEM-treated samples could be a consequence of IL-1β cleavage inhibition. The active form of this cytokine acts as a positive autocrine signal to induce a feedback loop of proIL-1β expression [32].

Inflammasome activation also produces pyroptosis and, consequently, the release of lactate dehydrogenase (LDH) to media. However, VX-765, the inhibitor of caspase-1, does not affect pyroptosis in THP-1 cells. Interestingly, MEM treatment reduces the release of LDH. Thus MEM is able to partially inhibit pyroptosis produced by inflammasome activation (Fig. 3d).

**Fig. 4:**
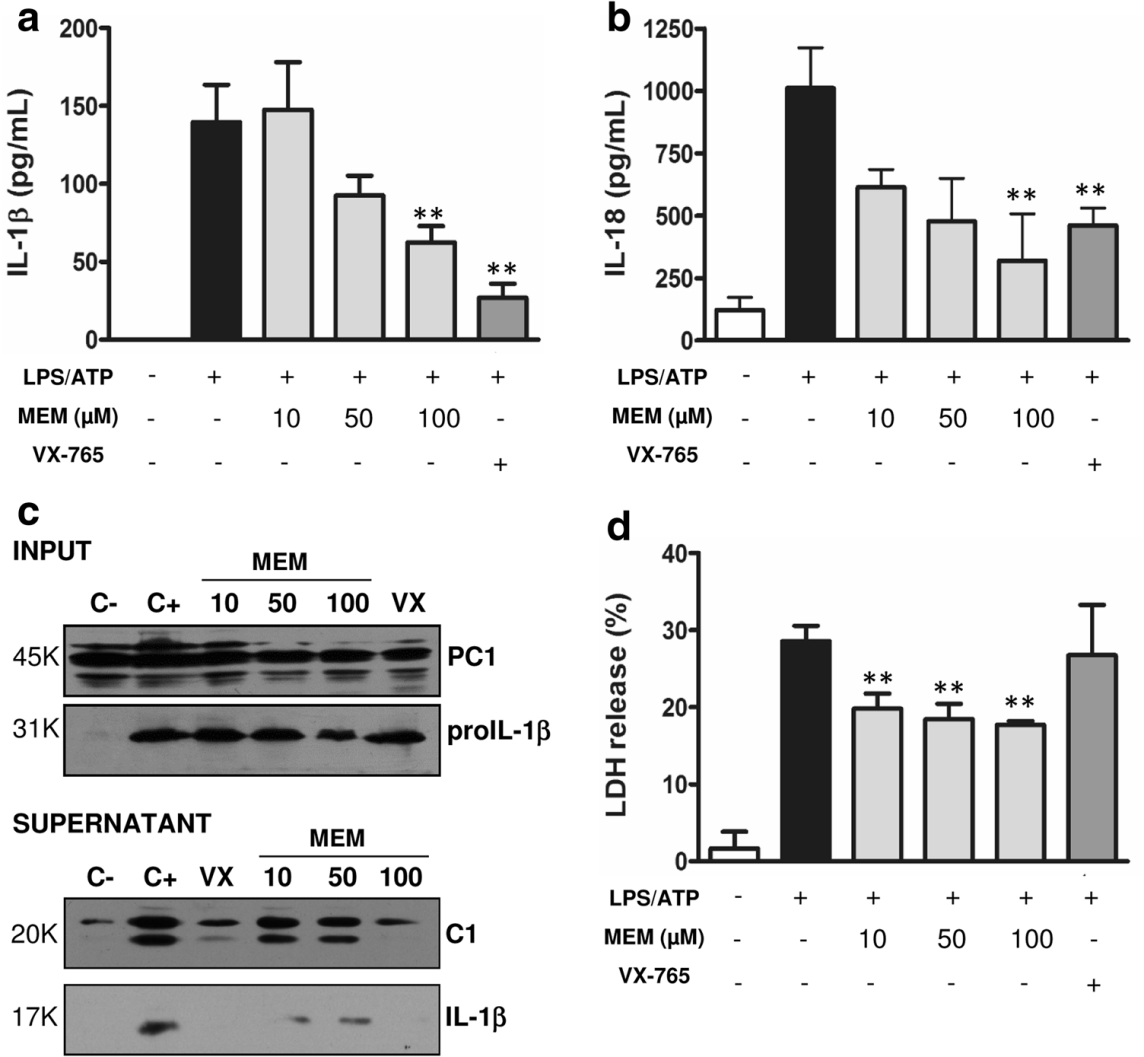
MEM inhibits the NLRP3-dependent inflammasome in THP-1 cells. IL-1β (**a**) and IL-18 (**b**) secretion was evaluated by the ELISA technique upon activation of the NLRP3 inflammasome with LPS (100 ng/ml) and ATP (2.5 mM). Cells were treated with MEM at 10, 50 or 100 µM, and with VX-765 (VX) at 5 µM. **c** THP-1 cells were stimulated as described above, and supernatants and inputs were analyzed by Western blotting; IL-1β and cleaved caspase-1 (C1) were detected. The shown data are representative of three independent experiments. **d** Measurement of the release of LDH into the extracellular medium under the above-described conditions. C−: cells non-treated and C+: cells treated with stimuli activation but without the anti-inflammatory treatment. *Asterisks* represent significant differences to the positive control (C+), as determined by a one-way ANOVA test with Dunnetts’s multiple *post test* comparison **p < 0.05. All the data are expressed as the mean ± SD of three experiments

In order to activate the NLRP3 inflammasome, a combination of LPS and adenosine triphosphate (ATP) stimuli was used [31]. Inhibition of IL-1β and IL-18 secretion by MEM gave similar results as in the case of NLRP1 (Fig. 4a, b). Immunoblotting experiments showed that proIL-1β decreased at the highest MEM concentration [32], as well as diminished caspase-1 and IL-1β processing (Fig. 4c and Supp data 1). The analysis of cellular pyroptosis by the release of LDH to media showed similar results to the NLRP1 inflammasome activation study. A lower cytotoxicity was observed in the MEM-treated cells than in the VX-765-treated cells (Fig. 4d), which indicates that MEM protects cells from pyroptotic damage.

## Discussion

MEM (or methergine) is a synthetic analog of ergonovine, a psychedelic alkaloid found in ergot. It is a smooth muscle constrictor that functions via the antagonism of the dopamine D1 receptor which mostly acts on the uterus [33, 34]. To this end, it is widely used in obstetrics to prevent or control excessive bleeding following childbirth and abortion. This study shows that MEM also exerts an inhibitory effect on PC1 activation, a key protein in the inflammation process, in vitro and cell-based assays with an IC_50_ within the micromolar range. Similar results were obtained in both the NLRP1 and NLRP3 inflammasome activation models.

It is noteworthy that MEM is also able to significantly reduce the release of LDH caused by inflammasome activation in THP-1 cells. Compound VX-765 is the most effective inhibitor of caspase-1 as it acts on the catalytic site of the enzyme [27], but does not decrease pyroptosis in THP-1 cells. MEM is able to inhibit pyroptosis and enhance cell recovery. This result supports the hypothesis that targeting ASC-mediated PC1 activation is an advantage for cell protection over active caspase-1 inhibition. In line with our data, recent research has given rise to the development of a single domain antibody fragment that specifically recognizes the CARD domain of human ASC [35]. This antibody impairs ASC (CARD) interactions, inhibits inflammasome activation and, more importantly, protects cells from inflammatory cell death. Our results reinforce the idea that the perturbation of ASC interaction interfaces could be a potential target for the development of broad-spectrum anti-inflammatory agents with improved cell recovery capabilities.

MEM is the first pass metabolite of the methysergide maleate drug [36]. The anti-inflammatory activity of methysergide maleate has been reported in systemic burn edema, asthma and endotoxemia animal models, but the mechanism of action has not been thoroughly explored [37–39]. In these models, the contribution of the inflammasome has been recently reported [40, 41]. Although further research will be required to clarify the in vivo anti-inflammatory activity of MEM, our results suggest the contribution of inflammasome inhibition to the anti-inflammatory effects observed in asthma and endotoxemia models.

## Materials and methods

### Purification of procaspase-1 (PC1)

Human His-tagged PC1 was previously cloned in the pFastBac vector for Baculovirus expression. The expression plasmid was transformed into DH10Bac *Escherichia coli* cells. Recombinant bacmids were purified and used to transfect Sf9 insect cells. The virus stock was amplified and used to infect suspension cultures. Briefly, Sf9 cells were grown in 2 L of Grace’s Insect Medium (Gibco) and infected at 1,500,000 cells/mL with a recombinant virus that contained His-tagged PC1 at a multiplicity of infection of 1 (MOI 1). Then they were cultured for 16 h at 27 °C with constant gentle agitation to express the protein. Cells were centrifuged at 1000 g for 10 min at 4 °C and washed with PBS. The pellet was lysed with buffer lysis (100 mM Hepes-KOH, pH 7.5, 50 mM KCl, 7.5 mM MgCl_2_, 5 mM NaEDTA, 5 mM NaEGTA and proteases inhibitors: Pepstatin, Leupeptin and PMSF) using a Douncer and clarified by centrifugation (10,000 g, 1 h at 4 °C). The resulting supernatant that contained the recombinant protein was purified in an Ni-NTA (Ni2þ-nitrilotriacetate)-agarose column. After elution, PC1 was further purified by ion exchange chromatography (Mono Q) in 20 mM of Hepes-KOH, pH 7.5, 10 mM KCl, 1.5 mM MgCl_2_, 1 mM EDTA, 1 mM EGTA.

### Purification of the apoptosis-associated speck-like protein (ASC)

Human ASC was previously cloned in the peT28a vector for its expression in *E. coli* cells. Briefly, 8 L of LB medium, plus antibiotics chloramphenicol and kanamycin, were inoculated with an overnight culture of freshly transformed His tagged-ASC-pet28a-BL21 (DE3) pLys *E. coli* cells (Invitrogen), and grown at 37 °C until OD 0.7. The ASC expression was induced with 0.7 mM IPTG at 28 °C for 4 h. Cells were harvested (15 min, 6000 rpm at 4 °C), lysed in 300 mL of Buffer A (50 mM NaH_2_PO_4_, 300 mM NaCl, pH 8.0) and sonicated. After centrifugation at 11,000 rpm for 1 h at 4 °C, ASC was present in the inclusion bodies (pellet). Pellets were then resuspended in 100 mL of denaturing Buffer B (50 mM NaH_2_PO_4_, 300 mM NaCl, 8 M urea, pH 8.0) and resonicated. Under these conditions, the His tagged-ASC protein was solubilized and purified using TALON resin (Qiagen). Re-folding was performed in the TALON resin at a gradient of decreasing urea concentrations and the protein was finally eluted in 15 mL of Buffer C (50 mM NaH_2_PO4, 300 mM NaCl, 500 mM imidazol, pH 8.0). Desalting was performed in a PD-10 column and the protein was finally concentrated in a 10K Amicon concentrator unit.

### Primary screening: the ASC-mediated PC1 reconstitution in vitro assay

Recombinant PC1 (75 nM) was pre-incubated with the indicated concentrations of compounds for 20 min at RT in Assay Buffer (20 mM Hepes pH 7.5, 10 mM KCl, 1.5 mM MgCl_2_, 1 mM EDTA, 1 mM EGTA, 1 mM DTT). Then 300 nM of ASC were added and incubated for another 10-min period. Caspase-1 activation was measured using fluorogenic substrate Ac-WEHD-AFC (80 µM; PeptaNova) by continuously monitoring the release of the AFC product in a Wallac Victor 1420 workstation. The Prestwick Chemical Library^®^ is composed of 880 FDA-approved drugs dissolved in DMSO. The compounds were initially tested at 20 µM with 1% of DMSO. Data are reported as the percentage of inhibition compared with the absence of the compound.

### Secondary screening: the caspase-1 in vitro activation assay

Recombinant caspase-1 (75 nM) was incubated with the indicated concentrations of compounds for 20 min at RT in Assay Buffer. Then fluorogenic substrate Ac-WEHD-AFC (80 µM) was added and activity was monitored in a Wallac Victor 1420 workstation. Data are reported as the percentage of inhibition compared with the absence of the inhibitor.

### Toxicity assay: the MTS (3-(4,5-dimethylthiazol-2-yl)-5-(3-carboxymethoxyphenyl)-2-(4-sulfophenyl)-2H-tetrazolium, inner salt) assay

THP-1 is a cell line that is derived from human acute monocytic leukemia purchased from ATCC. THP-1 cells were cultured in RPMI supplemented with 10% FBS (Sigma) at 37 °C and 5% CO_2_.

Cell proliferation was measured with the CellTiter 96^®^ Aqueous Non-Radioactive Cell Proliferation Assay kit (Promega) according to the manufacturer’s instructions. THP-1 cells were plated in 96-well plates at a cellular density of 10,000 cells/well. Cells were treated with the indicated compounds at five concentrations for 24 h. Plates were read at 490 nm by a Wallac Victor 1420 workstation.

### The inflammasome activation cellular assays

The molecules were evaluated in the THP-1 cells stimulated with LPS and MDP, or with LPS plus ATP, to stimulate ASC-dependent inflammasomes NLRP1 and NLRP3, respectively. Briefly, 1 × 10^6^ cells were seeded in 6-well plates in 1 mL RPMI media that contained 1% FBS. Cells were either mock-treated or primed with compounds at the indicated concentrations for 30 min, followed by treatment with 100 ng/mL LPS (Sigma) and 50 μg/mL MDP (Sigma) or 2.5 mM ATP (Sigma) for 6 h at 37 °C. Supernatants were harvested and clarified by centrifugation at 1500 rpm at RT and a cytokine analysis was performed.

Both IL-1β secretion and the release of IL-18 were monitored by an ELISA assay (BD OptEIA™ Human IL-1β ELISA Kit and Human IL-18 Module Set eBioscience) following the manufacturer’s instructions. Cell viability was analyzed in parallel by evaluating the release of LDH according to a commercial kit (CytoTox-ONE™ Homogeneous Membrane Integrity Assay; Promega). The release of LDH was calculated using the formula: release of LDH (%) = 100 × (Abs490 treated − Abs490 untreated cells)/Abs490 untreated cells lysed with Triton 9% (maximum release of LDH).

### Immunoblotting

The supernatants of the treated cells were precipitated by the chloroform–methanol method, as described by De Nardo and Latz [42]. Pellets were obtained by lysing cells in 25 mM of Tris-HCl, pH 7.4, 1 mM EDTA, 1 mM EGTA and 1% SDS, plus protease and phosphatase inhibitors. The protein concentration was determined by the BCA protein assay. Samples were separated in a 14% SDS-PAGE gel, transferred to a nitrocellulose membrane and blocked with 5% skimmed milk for 1 h. Then the membrane was incubated overnight with primary antibodies: α-casp1 (1:1000; Cell Signaling #2225) and α-IL-1β (1:1000; Acris #R1130P) at 4 °C. Membranes were washed and probed with the appropriate secondary antibody conjugated with peroxidase for enhanced chemiluminescence detection (Amersham Pharmacia Biotech).

### Statistical analysis

Data were analyzed with the GraphPad software and statistical significance was assessed by a Student’s *t*-test or a one-way analysis of variance (ANOVA) test with Dunnetts’s multiple *post test* comparison (**p < 0.05; ***p < 0.01).

## Electronic supplementary material

Below is the link to the electronic supplementary material.

Supplementary material 1 (PDF 222 KB)
