# Supplementary material for: Identification and validation of uterine stimulant methylergometrine as a potential inhibitor of caspase-1 activation
Source: Apoptosis. 2017 Jul 28;22(10):1310–8. doi: 10.1007/s10495-017-1405-z (PMC5630661; doi:10.1007/s10495-017-1405-z)
Supplement: Supplementary file 1 — Supplementary material 1 (PDF 222 KB) [file 10495_2017_1405_MOESM1_ESM.pdf]

Supp Data 1

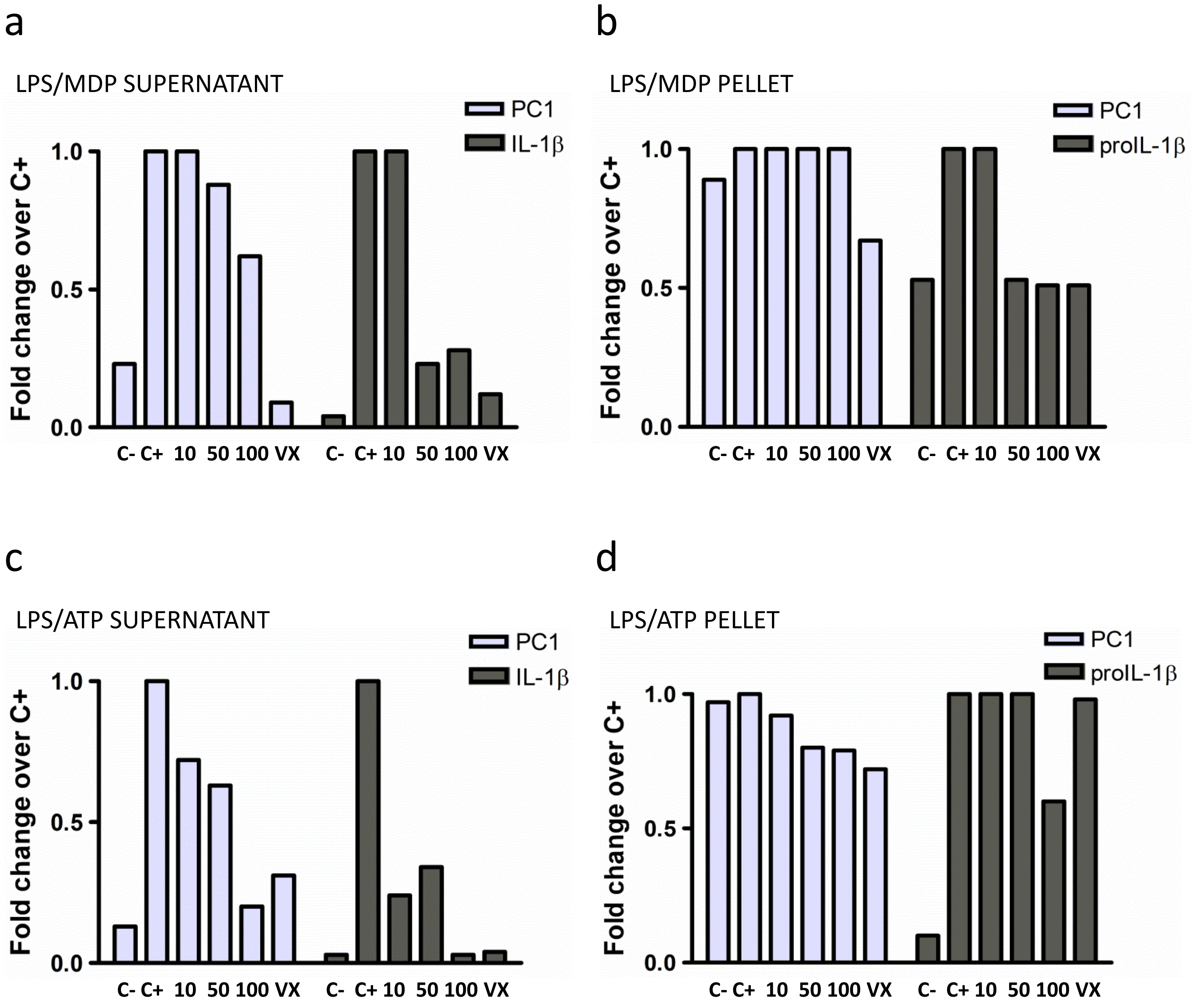

Supp data 1 Densitometric analyses of immunoblots from Fig. 3c and Fig. 4c. Bands were quantified using the Image J software and reported as arbitrary units (a.u).
